# Cross-sectional study of associations between normal body weight with central obesity and hyperuricemia in Japan

**DOI:** 10.1186/s12902-019-0481-1

**Published:** 2020-01-06

**Authors:** Takako Shirasawa, Hirotaka Ochiai, Takahiko Yoshimoto, Satsue Nagahama, Akihiro Watanabe, Reika Yoshida, Akatsuki Kokaze

**Affiliations:** 1 Department of Hygiene, Public Health and Preventive Medicine, Showa University School of Medicine, 1-5-8 Hatanodai, Shinagawa-ku, Tokyo, 142-8555 Japan; 2 All Japan Labor Welfare Foundation, 6-16-11 Hatanodai, Shinagawa-ku, Tokyo, 142-0064 Japan

**Keywords:** Normal weight central obesity, Body mass index, Waist-to-height ratio, Hyperuricemia

## Abstract

**Background:**

Several studies have shown that normal weight with central obesity (NWCO) is associated with cardiovascular disease risk factors such as hypertension, dyslipidemia and diabetes. However, the relationship between NWCO and hyperuricemia has not been studied in detail.

**Methods:**

We investigated the association between NWCO and hyperuricemia among Japanese adults aged 40–64 years who had undergone periodic health examinations between April 2013 and March 2014. Obesity was defined as a body mass index (BMI) ≥25 kg/m^2^ and central obesity was determined as a waist-to-height ratio (WHtR) ≥0.5. We classified the participants into the following groups based according to having obesity and central obesity: normal weight (BMI 18.5–24.9 kg/m^2^) without (NW; WHtR < 0.5) and with (NWCO) central obesity, and obesity without (OB) and with (OBCO) central obesity. Hyperuricemia was defined as serum uric acid > 7.0 and ≥ 6.0 mg/dL in men and women, respectively, or under medical treatment for hyperuricemia. Alcohol intake was classified as yes (daily and occasional consumption) and none (no alcohol consumption). Odds ratios (OR) and 95% confidence intervals (CI) for hyperuricemia were calculated using a logistic regression model.

**Results:**

We analyzed data derived from 96,863 participants (69,241 men and 27,622 women). The prevalences of hyperuricemia in men and women were respectively, 21.4 and 11.0%, and of participants with NWCO respectively 15.6 and 30.0%. The adjusted OR for hyperuricemia was significantly increased in OBCO compared with NW, regardless of sex (men: OR, 2.12; 95%CI; 2.03–2.21; women: OR, 3.54; 95%CI, 3.21–3.90) and were statistically significant in NWCO compared with NW (men: OR, 1.44; 95%CI, 1.36–1.52; women: OR, 1.41; 95%CI, 1.27–1.57). The results were similar regardless of alcohol consumption.

**Conclusions:**

We found that NWCO and OBCO were associated with hyperuricemia in middle-aged Japanese men and women. Middle-aged Japanese adults with normal weight but having central obesity should be screened using a combination of BMI and WHtR and educated about how to prevent hyperuricemia.

## Background

Hyperuricemia is associated with increased risk of gout [1], hypertension, chronic kidney diseases, congestive heart failure, metabolic syndrome, type 2 diabetes mellitus and cardiovascular disease (CVD) [2]. Therefore, the prevention of hyperuricemia is an important public health issue.

Several studies have associated normal weight with central obesity (NWCO), defined by body mass index (BMI) and waist-to-height ratio (WHtR), and the CVD risk factors of hypertension, dyslipidemia and diabetes [3, 4]. In addition, NWCO is associated with a significantly higher risk for metabolic syndrome [5]. Thus, measuring degrees of central fat distribution appear important for the early detection of health risks, even among individuals with normal weight (NW) [6]. However, relationships between NWCO and hyperuricemia remain obscure.

The present study aimed to determine relationships between NWCO and hyperuricemia among Japanese middle-aged adults. We postulated that Japanese men and women are at higher risk for hyperuricemia when they have NWCO, than NW.

## Methods

### Participants

The present study was a cross-sectional study that used the data (*n* = 310,577) from Japanese men and women who had underwent periodic health examinations conducted by the All Japan Labor Welfare Foundation (Tokyo), between April 2013 and March 2014. The inclusion criteria were individuals aged 40–64 years and those who consented to participate in this study. The exclusion criteria were those who had missing data and who were underweight (BMI < 18.5 kg/m^2^).

Of 310,577, 310,498 individuals consented to participate in this study, we excluded 205,730 with missing data and 7905 who were underweight. Thus, we analyzed data from 96,863 participants (69,241 men and 27,622 women).

Written informed consent was obtained from the included individuals to participate in the study and to publish their innominate data. The Medical Ethics Committee at Showa University School of Medicine (Approval No. 2132) and the Ethics Committee at the All Japan Labor Welfare Foundation (Approval No. 3-1-0004) approved the study protocol.

### Variables and measurements

We collected the following information from each participant using a self-administered questionnaire recommended by the Japanese Ministry of Health, Labour and Welfare for specific health examinations [7]: age, sex, alcohol consumption (daily, occasionally, none), smoking status (current, previously smoked, never-smoked), and physical activity equivalent to walking at least 60 min per day (yes/no). Alcohol intake was classified as yes (daily and occasional consumption) and no (no alcohol consumption) [8].

Trained staff measured the height and weight of the participants in increments of 0.1 cm and 0.1 kg, respectively, and BMI was calculated as body weight (kg) divided by height squared (m^2^). Waist circumference (WC) was measured to the nearest 0.1 cm at the level of the umbilicus while standing upright [6]. The WHtR was calculated as WC divided by height. Blood pressure while seated was measured using a HEM-907 automated device (Omron Corporation, Kyoto, Japan).

In accordance with a previous study [9], we defined OB and NW defined as BMI ≥25 and 18.5–24.9 kg/m^2^, respectively. In addition, the presence and absence of CO were determined as WHtR ≥0.5 and <  0.5, respectively [10]. Based on OB and CO status, participants in this study were classified as being of normal weight with (NWCO) and without (NW) CO, and as being obese with (OBCO) and without (OB) central obesity [11]. Hypertension was defined as systolic blood pressure ≥ 140 mmHg, diastolic blood pressure ≥ 90 mmHg, or under medication for hypertension [12].

Venous blood samples with drawn from participants to determine serum values of uric acid, high-density lipoprotein cholesterol (HDL-C), low-density lipoprotein cholesterol (LDL-C), triglyceride, blood glucose, and hemoglobin A1c (HbA1c) were stored at 4 °C, transported to and analyzed at a clinical testing laboratory (SRL Inc., Tokyo, Japan) within 24 h.

Serum uric acid was measured using an enzymatic method (AU5400; Beckman Coulter, Brea, CA, USA). Although there was a need to define hyperuricemia in relation to increase uric acid level either by overproduction or due to decrease secretion, in the present study, hyperuricemia was defined as serum uric acid > 7.0 mg/dL in men or ≥ 6.0 mg/dL in women, or being under medical treatment for hyperuricemia, which was based solely on serum uric acid levels [13–16]. Serum uric acid levels are lower in women than in men because female hormones decrease them [17, 18]. These cutoff values were selected as they are generally used in clinical laboratories and have been proposed in previous studies in relation to metabolic syndrome and CVD outcomes to define hyperuricemia [13–16, 19]. Both HDL-C and LDL-C were determined using a direct method and triglycerides were measured using an enzymatic method (AU5400; Beckman Coulter). Dyslipidemia was defined as LDL-C ≥ 140 mg/dL, HDL-C < 40 mg/dL, triglyceride ≥150 mg/dL, or under medication for dyslipidemia [20]. Blood glucose values were determined using the hexokinase method (AU5400; Beckman Coulter), and HbA1c was measured using a latex agglutination method (JCA-BM9130; JEOL, Tokyo, Japan). Diabetes was defined as fasting plasma glucose (≥8 h after the last caloric intake) ≥126 mg/dL, random plasma glucose ≥200 mg/dL, HbA1c (National Glycohemoglobin Standardization Program) ≥6.5%, or under medication for diabetes mellitus [21].

### Statistical analysis

Data for males and females were separately analyzed, because the serum uric acid distribution differed between them. Characteristics were compared between participants with and without hyperuricemia using unpaired t-tests or chi-squared tests. Odds ratios (OR) and 95% confidence intervals (CI) for hyperuricemia were calculated using a logistic regression model that included age, smoking status, physical activity, hypertension, dyslipidemia, and diabetes to control for potential confounding factors [11, 16]. Values with *P* <  0.05 were considered statistically significant. All data were analyzed using JMP version 13.0 (SAS Institute Japan Co., Ltd., Tokyo, Japan).

## Results

Table 1 shows the characteristics of study participants with mean ages of 50.4 and 50.6 years for men and women, respectively. Means serum uric acid values and the prevalence of hyperuricemia were higher in men than in women (6.1 vs. 4.6 mg/dL and 21.4% vs. 11.0%, respectively). The proportions of NWCO among men and women were 15.6 and 30.0%, respectively, and those of men and women who consumed alcohol were 73.2 and 46.3%, respectively.

**Table 1:** Characteristics of participants

	Men (N=69,241)	Women (N=27,622)
Age (y)	50.4 (7.1)	50.6 (6.9)
Height (cm)	169.8 (6.3)	157.7 (6.3)
Weight (kg)	69.3 (11.0)	57.1 (9.8)
BMI (kg/m^2^)	24.0 (3.3)	22.9 (3.5)
WC (cm)	84.8 (9.0)	80.5 (9.2)
WHtR	0.50 (0.05)	0.51 (0.06)
Obesity types		
NW	36,069 (52.1)	13,189 (47.7)
NWCO	10,794 (15.6)	8,276 (30.0)
OB	1,458 (2.1)	107 (0.4)
OBCO	20,920 (30.2)	6,050 (21.9)
Uric acid (mg/dL)	6.1 (1.3)	4.6 (1.1)
Hyperuricemia	14,823 (21.4)	3,033 (11.0)
Hypertension	25,911 (37.4)	8,312 (30.1)
Dyslipidemia	37,557 (54.2)	11,918 (43.1)
Diabetes	6,226 (9.0)	1,123 (4.1)
Smoking status		
Current	29,867 (43.1)	5,032 (18.2)
Quit	13,868 (20.0)	2,043 (7.4)
Never smoked	25,506 (36.8)	20,547 (74.4)
Physical activity		
Yes	22,973 (33.2)	8,207 (29.7)
No	46,268 (66.8)	19,415 (70.3)
Alcohol intake		
Yes	50,624 (73.2)	12,784 (46.3)
No	18,617 (26.9)	14,838 (53.7)

Data are expressed as means (standard deviation) or n (%)

*BMI* body mass index, *NW* normal weight without central obesity, *NWCO* normal weight with central obesity, *OB* obesity without central obesity, *OBCO* obesity with central obesity, *WC* waist circumference, *WHtR* waist-to-height ratio

Tables 2 and 3 respectively show the characteristics of men and women with hyperuricemia and normouricemia. Differences were statistically significant between the nature of the obesity and hyperuricemia in men and women. The prevalences of OBCO in hyperuricemia and normouricemia were 43.3 and 26.7% among men, and 44.8 and 19.1% among women. The prevalences of hypertension were 45.2 and 35.3% in men with hyperuricemia and normouricemia, respectively, and 43.8 and 28.4% in women with hyperuricemia and normouricemia, respectively. Those of dyslipidemia were 66.0 and 51.1% in men with hyperuricemia and normouricemia, and 60.7 and 41.0% in women with hyperuricemia and normouricemia. The prevalences of diabetes among men with hyperuricemia and normouricemia were 7.2 and 9.5%, while those among women with hyperuricemia and normouricemia were 8.0 and 3.6%. Moreover, the proportions of those who habitually consumed alcohol were higher in both men and women with, than without hyperuricemia.

**Table 2:** Characteristics of male participants

	Hyperuricemia (N=14,823)	Normouricemia (N=54,418)	*P*^a^
Age (y)	50.0 (6.9)	50.6 (7.1)	< 0.001
Height (cm)	170.3 (5.9)	169.6 (6.3)	< 0.001
Weight (kg)	73.0 (11.8)	68.3 (10.6)	< 0.001
BMI (kg/m^2^)	25.1 (3.6)	23.7 (3.2)	< 0.001
WC (cm)	87.9 (9.2)	83.9 (8.7)	< 0.001
WHtR	0.52 (0.05)	0.50 (0.05)	< 0.001
Obesity types			
NW	5,722 (38.6)	30,347 (55.8)	< 0.001
NWCO	2,366 (16.0)	8,428 (15.5)	
OB	321 (2.2)	1,137 (2.1)	
OBCO	6,414 (43.3)	14,506 (26.7)	
Uric acid (mg/dL)	7.8 (0.8)	5.6 (0.9)	< 0.001
Hypertension	6,694 (45.2)	19,217 (35.3)	< 0.001
Dyslipidemia	9,779 (66.0)	27,778 (51.1)	< 0.001
Diabetes	1,073 (7.2)	5,153 (9.5)	< 0.001
Smoking status			
Current	5,915 (39.9)	23,952 (44.0)	< 0.001
Quit	3,508 (23.7)	10,360 (19.0)	
Never smoked	5,400 (36.4)	20,106 (37.0)	
Physical activity			
Yes	4,647 (31.4)	18,326 (33.7)	< 0.001
No	10,176 (68.7)	36,092 (66.3)	
Alcohol intake			
Yes	11,860 (80.0)	38,764 (71.2)	< 0.001
No	2,963 (20.0)	15,654 (28.8)	

Data are expressed as means (standard deviation) or n (%)

*BMI* body mass index, *NW* normal weight without central obesity, *NWCO* normal weight with central obesity, *OB* obesity without central obesity, *OBCO* obesity with central obesity, *WC* waist circumference, *WHtR* waist-to-height ratio

^a^Unpaired t-test or chi-squared test

**Table 3:** Characteristics of female participants

	Hyperuricemia (N=3,033)	Normouricemia (N=24,589)	*P*^a^
Age (y)	51.9 (7.0)	50.4 (6.9)	< 0.001
Height (cm)	160.3 (8.4)	157.4 (5.9)	< 0.001
Weight (kg)	65.0 (12.7)	56.2 (8.9)	< 0.001
BMI (kg/m^2^)	25.2 (4.3)	22.6 (3.3)	< 0.001
WC (cm)	86.9 (10.2)	79.7 (8.7)	< 0.001
WHtR	0.54 (0.07)	0.51 (0.06)	< 0.001
Obesity types			
NW	826 (27.3)	12,363 (50.3)	< 0.001
NWCO	823 (27.2)	7,453 (30.3)	
OB	24 (0.8)	83 (0.3)	
OBCO	1,360 (44.8)	4,690 (19.1)	
Uric acid (mg/dL)	6.7 (0.7)	4.3 (0.8)	< 0.001
Hypertension	1,327 (43.8)	6,985 (28.4)	< 0.001
Dyslipidemia	1,840 (60.7)	10,078 (41.0)	< 0.001
Diabetes	241 (8.0)	882 (3.6)	< 0.001
Smoking status			
Current	799 (26.3)	4,233 (17.2)	< 0.001
Quit	396 (13.1)	1,647 (6.7)	
Never smoked	1,838 (60.6)	18,709 (76.1)	
Physical activity			
Yes	868 (28.6)	7,339 (29.9)	0.163
No	2,165 (71.4)	17,250 (70.2)	
Alcohol intake			
Yes	1,780 (58.7)	11,004 (44.8)	< 0.001
No	1,253 (41.3)	13,585 (55.3)	

Data are expressed as means (standard deviation) or n (%)

*BMI* body mass index, *NW* normal weight without central obesity, *NWCO* normal weight with central obesity, *OB* obesity without central obesity, *OBCO* obesity with central obesity, *WC* waist circumference, *WHtR* waist-to-height ratio

^a^Unpaired t-test or chi-squared test

Tables 4 and 5 show the crude and adjusted OR and 95%CI for hyperuricemia among men and women, respectively. The adjusted OR for hyperuricemia was significantly increased in men and women with OBCO compared with NW after adjustment for age, lifestyle factors, hypertension, dyslipidemia, and diabetes (OR, 2.12; 95%CI, 2.03–2.21 and OR, 3.54; 95%CI, 3.21–3.90, respectively). The OR for hyperuricemia were also significantly increased in men and women with NWCO compared with NW (OR, 1.44; 95%CI, 1.36–1.52 and OR, 1.41; 95%CI, 1.27–1.57, respectively). An analysis of subgroups stratified by alcohol intake found that the OR for hyperuricemia was significantly increased among men and women with OBCO who consumed alcohol (OR, 1.95; 95%CI, 1.86–2.05 and OR, 2.94; 95%CI, 2.59–3.35, respectively) and among those who did not (OR, 3.27; 95%CI, 2.97–3.60 and OR, 5.29; 95%CI, 4.50–6.22, respectively) compared with NW. The OR for hyperuricemia were statistically significant among men and women with NWCO who consumed alcohol (OR, 1.42; 95%CI, 1.34–1.51 and OR, 1.25; 95%CI, 1.09–1.42, respectively) compared with those who did not consume alcohol (OR, 1.56: 95%CI, 1.36–1.78 and OR, 1.85; 95%CI, 1.55–2.21, respectively). These results persisted even after adjustment for age, lifestyle factors, hypertension, dyslipidemia, diabetes, and the estimated glomerular filtration rate.

**Table 4:** Odds ratios and 95% confidence intervals for hyperuricemia in men

		Total	HU	Crude	Adjusted
		n	n (%)	OR 95%CI	OR 95%CI
Total (N=69,241)	NW	36,069	5,722 (15.9)	1	1
	NWCO	10,794	2,366 (21.9)	1.49 (1.41-1.57)	1.44 (1.36-1.52)
	OB	1,458	321 (22.0)	1.50 (1.32-1.70)	1.33 (1.17-1.51)
	OBCO	20,920	6,414 (30.7)	2.35 (2.25-2.44)	2.12 (2.03-2.21)
Alcohol consumption					
Yes (n=50,624)	NW	26,941	4,897 (18.2)	1	1
	NWCO	8,046	1,978 (24.6)	1.46 (1.38-1.56)	1.42 (1.34-1.51)
	OB	1,061	252 (23.8)	1.40 (1.21-1.62)	1.23 (1.06-1.42)
	OBCO	14,576	4,733 (32.5)	2.16 (2.07-2.27)	1.95 (1.86-2.05)
No (n=18,617)	NW	9,128	825 (9.0)	1	1
	NWCO	2,748	388 (14.1)	1.65 (1.45-1.88)	1.56 (1.36-1.78)
	OB	397	69 (17.4)	2.12 (1.62-2.77)	1.94 (1.48-2.54)
	OBCO	6,344	1,681 (26.5)	3.63 (3.31-3.97)	3.27 (2.97-3.60)

Adjusted for age, smoking status, physical activity, hypertension, dyslipidemia and diabetes

*CI* confidence interval, *HU* hyperuricemia, *NW* normal weight without central obesity, *NWCO* normal weight with central obesity, *OB* obesity without central obesity, *OBCO* obesity with central obesity, *OR* odds ratio

**Table 5:** Odds ratios and 95% confidence intervals for hyperuricemia in women

		Total	Cases	Crude	Adjusted
		n	n (%)	OR 95%CI	OR 95%CI
Total (N=27,622)	NW	13,189	826 (6.3)	1	1
	NWCO	8,276	823 (9.9)	1.65 (1.49-1.83)	1.41 (1.27-1.57)
	OB	107	24 (22.4)	4.33 (2.73-6.85)	3.76 (2.34-6.02)
	OBCO	6,050	1,360 (22.5)	4.34 (3.96-4.76)	3.54 (3.21-3.90)
Alcohol consumption					
Yes (n=12,784)	NW	6,467	597 (9.2)	1	1
	NWCO	3,752	491 (13.1)	1.48 (1.30-1.68)	1.25 (1.09-1.42)
	OB	60	21 (35.0)	5.29 (3.09-9.06)	4.63 (2.66-8.08)
	OBCO	2,505	671 (26.8)	3.60 (3.18-4.06)	2.94 (2.59-3.35)
No (n=14,838)	NW	6,722	229 (3.4)	1	1
	NWCO	4,524	332 (7.3)	2.25 (1.89-2.67)	1.85 (1.55-2.21)
	OB	47	3 (6.4)	1.93 (0.60-6.27)	1.69 (0.51-5.54)
	OBCO	3,545	689 (19.4)	6.84 (5.85-7.99)	5.29 (4.50-6.22)

Adjusted for age, smoking status, physical activity, hypertension, dyslipidemia and diabetes

*CI* confidence interval, *HU* hyperuricemia, *NW* normal weight without central obesity, *NWCO* normal weight with central obesity, *OB* obesity without central obesity, *OBCO* obesity with central obesity, *OR* odds ratio

## Discussion

The present study investigated the relationships between NWCO and hyperuricemia in middle-aged Japanese adults. We found that hyperuricemia was significantly associated with NWCO and OBCO, compared with men and women of NW. The results were similar regardless of alcohol consumption.

The mean serum uric acid value was higher in men than in women. Several studies have investigated serum uric acid levels in Japanese adults. Reported serum uric acid levels for men and women are respectively, 6.2 and 4.4 mg/dL [14], 5.9 and 4.2 mg/dL [15], 6.5 and 4.7 mg/dL [16], and 6.0 and 4.5 mg/dL [22]. Therefore, our findings of higher serum acid levels in men were similar to these findings.

Several epidemiological studies reported that hyperuricemia was associated with diseases including diabetes mellitus, dyslipidemia, hypertension, CVD, and metabolic syndrome [2, 8, 14, 16]. Similarly, the present study showed that the prevalences of hypertension, dyslipidemia, and diabetes were higher with than without hyperuricemia in both men and women. Moreover, we also found that OBCO significantly increased the OR for hyperuricemia, compared with NW, regardless of sex. Others have positively associated hyperuricemia with obesity in both men and women [16, 23]. Nagahama et al. reported that the adjusted OR (95%CI) for hyperuricemia in men and women with BMI ≥25 kg/m^2^ were 1.75 (1.56–1.97) and 2.02 (1.62–2.53) [16]. Yu et al. reported that hyperuricemia was related to abdominal obesity and general obesity in both sexes [23]. The present results are in line with these findings. Even after adjustment for age, lifestyle factors, hypertension, dyslipidemia, and diabetes, our results showed that OBCO had an independently increased risk of hyperuricemia and this was statistically significant between OBCO and hyperuricemia. As one of the reasons, obesity may increase hyperuricemia through increased urate production and decreased renal clearance, and that renal excretion of urate was also reduced in the presence of insulin resistance [24]. However, the underlying mechanism by which serum uric acid increased in obese individuals still remains to explore [19]. Thus, more studies need to be conducted in order to elucidate the mechanism of the association between serum uric acid and obesity.

In addition to OBCO, NWCO was also significantly associated with hyperuricemia compared with NW. A previous study in Japan found significantly increased OR (95%CI) for hyperuricemia in men with NWCO compared with NW, but not among women: 1.43 (1.15–1.76) and 2.20 (0.36–33.35), respectively [6]. We found that NWCO was associated with hyperuricemia risk compared with NW, regardless of sex. Moreover, WC and uric acid levels demonstrated a positive correlation in NW and NWCO group, regardless of sex (men, r = 0.19; *P* <  0.001, women, r = 0.23; *P* <  0.001). Higher and lower BMI are associated with an increased and decreased prevalence of hyperuricemia, respectively [8]. Therefore, because individuals with NWCO are considered to be of normal weight, they do not usually receive appropriate health education or prompt intervention to prevent hyperuricemia. Our findings suggest that men and women with NWCO need to be identified in addition to those with OBCO, even if their weight is normal.

Alcohol consumption is also an important risk factor for hyperuricemia [8, 25–27]. We stratified men and women according to alcohol intake and found that the OR for hyperuricemia were significantly increased in those with OBCO, compared with NW, regardless of alcohol consumption. That is, hyperuricemia was positively associated among men and women with OBCO, regardless of alcohol intake. Moreover, the OR for hyperuricemia were significantly increased in men and women with NWCO, compared with NW, regardless of whether alcohol was consumed. Therefore, screening men and women for NWCO and active intervention to prevent hyperuricemia are important, regardless of alcohol intake.

To the best of our knowledge, this is the first investigation into the relationships between hyperuricemia and NWCO among middle-aged Japanese adults. The strengths of the present study are the large sample size (about 100,000 participants), which helped to decrease random error, and fact that trained technicians measured the anthropometric variables of height, weight and WC of the participants, that were used to define OB and CO. Serum uric acid was measured using a standardized method at a clinical testing laboratory. In contrast, the limitations of the present study include potential confounding factors that were not determined in this study that might have affected our findings. For instance, we did not collect details of dietary intake such as the ingestion of large amounts of purine sources (animal protein and beer) [2]. Another limitation is that the cross-sectional study design caused difficulties with assessing causal relationships. For the nature of the study design, we did not observe these weight variations, although weight was very important clinical indicator in order to determine hyperuricemia throughout research in an individual. Thus, further longitudinal studies will be needed to establish causality and to consider weight variation.

## Conclusions

The present study showed that being of NW but having CO, and being obese in general were associated with hyperuricemia among middle-aged Japanese men and women. These findings suggest that middle-aged Japanese men and women with NWCO should be identified using a combination of BMI and WHtR, then educated about the prevention of hyperuricemia.

## Data Availability

The data in this study are available only upon reasonable request and approval by the Ethics Committee of the All Japan Labor Welfare Foundation.

## Abbreviations

BMI: Body mass index
CI: Confidence interval
CVD: Cardiovascular disease
HbA1c: Hemoglobin A1c
HDL-C: High-density lipoprotein cholesterol
LDL-C: Low-density lipoprotein cholesterol
NW: Normal weight without central obesity
NWCO: Normal weight with central obesity
OB: Obesity without central obesity
OBCO: Obesity with central obesity
OR: Odds ratio
WC: Waist circumference
WHtR: Waist-to-height ratio
