# Correction to: Cross-sectional study of associations between normal body weight with central obesity and hyperuricemia in Japan

**DOI:** 10.1186/s12902-020-0494-9

**Published:** 2020-02-21

**Authors:** Takako Shirasawa, Hirotaka Ochiai, Takahiko Yoshimoto, Satsue Nagahama, Akihiro Watanabe, Reika Yoshida, Akatsuki Kokaze

**Affiliations:** 1 Department of Hygiene, Public Health and Preventive Medicine, Showa University School of Medicine, 1-5-8 Hatanodai, Shinagawa-ku, Tokyo, 142-8555 Japan; 2 All Japan Labor Welfare Foundation, 6-16-11 Hatanodai, Shinagawa-ku, Tokyo, 142-0064 Japan

**Correction to: BMC Endocr Disord (2020) 20:2**

**https://doi.org/10.1186/s12902-019-0481-1**

After publication of this article [1], it was brought to our attention that there is an error in the Table 5, which “Cases” should be revised to “HU” in Table 5. The original publication has been corrected.
